# Self-compassion among Undergraduate Nursing Students at a State University in Indonesia during the COVID-19 Pandemic

**DOI:** 10.1590/0034-7167-2022-0585

**Published:** 2023-10-09

**Authors:** Khaira Ashfiya Salafi, Efri Widianti, Atlastieka Praptiwi

**Affiliations:** I Universitas Padjadjaran, Faculty of Nursing. Bandung, Indonesia

**Keywords:** COVID-19, Mental Health, Nursing Students, Self-Compassion, Self-Care, COVID-19, Saúde Mental, Estudantes de Enfermagem, Autocompaixão, Autocuidado, Infecciones por Coronavirus, Salud Mental, Estudiantes de Enfermería, Autocompasión, Autocuidado

## Abstract

**Objective:**

To determine the level of self-compassion and investigate the relationships between sociodemographic variables and self-compassion among undergraduate nursing students at a state university in Indonesia during the COVID-19 Pandemic.

**Methods:**

This study used a cross-sectional design. Samples were selected using a proportionate stratified random sampling technique (n=260). Data were collected using an Indonesian version of the Self-compassion Scale, which consists of 6 subscales: self-kindness, self-judgment, common humanity, isolation, mindfulness, and overidentification. Data were analyzed using univariate and bivariate analysis.

**Results:**

60% of students had moderate self-compassion. Students scored the highest in self-kindness (3,93±1,02) and over-identification (3,58±0,94), indicating that they often tried to love themselves when they felt emotional pain and often get carried away when something upsetting happened. Subsequently, a significant correlation was found between age and self-compassion (p<0.05).

**Conclusion:**

Self-compassion among nursing students should be improved through interventions such as compassion literacy, mindfulness training, and compassion-based experiential techniques.

## RESUMO

**Objetivo:**

Determinar o nível de autocompaixão e investigar as relações entre variáveis sociodemográficas e autocompaixão entre estudantes de enfermagem de uma universidade estadual da Indonésia durante a pandemia de COVID-19.

**Método:**

Este estudo utilizou um desenho transversal. As amostras foram selecionadas aplicando técnica de amostragem aleatória estratificada proporcional (n=260). Os dados foram coletados usando uma versão indonésia de Escala de Autocompaixão que consiste em 6 subescalas: auto bondade, autojulgamento, humanidade comum, isolamento, atenção plena e superidentificação. Os dados foram analisados por meio de análise univariada e bivariada.

**Resultados:**

60% dos alunos tinham autocompaixão moderada. Os alunos pontuaram mais em auto bondade (3,93±1,02) e superidentificação (3,58±0,94), o que indica que muitas vezes tentaram amar a si mesmos quando sentiram dor emocional e que, igualmente, deixaram-se levar quando algo perturbador aconteceu. Posteriormente, foi encontrada correlação significativa entre idade e autocompaixão (p<0,05).

**Conclusão:**

A autocompaixão entre estudantes de enfermagem deve ser melhorada por meio de intervenções, como grau de instrução em compaixão, treinamento em atenção plena e técnicas experienciais baseadas na compaixão.

## RESUMEN

**Objetivo:**

Determinar el nivel de autocompasión e investigar las relaciones entre las variables sociodemográficas y la autocompasión entre estudiantes de pregrado de enfermería en una universidad estatal de Indonesia durante la pandemia de COVID-19

**Métodos:**

Este estudio utilizó un diseño transversal. Las muestras se seleccionaron mediante una técnica de muestreo aleatorio estratificado proporcional (n=260). Los datos se recopilaron utilizando una versión indonesia de la Escala de Autocompasión, que consta de 6 subescalas: bondad propia, autocrítica, humanidad común, aislamiento, atención plena y sobre identificación. Los datos se analizaron mediante análisis univariado y bivariado.

**Resultados:**

El 60% de los estudiantes tenían autocompasión moderada. Los estudiantes puntuaron más alto en amabilidad consigo mismos (3,93±1,02) y sobre identificación (3,58±0,94), lo que indica que a menudo intentaban amarse a sí mismos cuando sentían dolor emocional y a menudo se dejaban llevar cuando sucedió algo perturbador. Posteriormente, se encontró una correlación significativa entre la edad y la autocompasión (p<0,05).

**Conclusión:**

La autocompasión entre los estudiantes de enfermería debe mejorarse por medio de intervenciones como la alfabetización de la compasión, el entrenamiento en atención plena y las técnicas experienciales basadas en la compasión.

## INTRODUCTION

The Corona Virus Disease 2019 (COVID-19) pandemic is the latest global health disaster of the century, with high rates of morbidity and mortality. Meanwhile, COVID-19 was declared a pandemic by World Health Organization (WHO) on March 11, 2020, with over 118,000 cases reported in 114 countries, and 4291 deaths^(1)^. In Indonesia, this disease was announced as a national disaster on April 20, 2020^(2)^. To suppress the spread, the Indonesian government encouraged all citizens to study and work from home^(3)^. As a result, nursing students experienced diverse educational challenges due to disrupted studies and modified learning strategies. Subsequently, online learning has limited clinical skill development in students, which is a common approach in the nursing curriculum. Virtual and adequate simulation equipment is not available in developing countries due to limited access and costs^(4)^, leading to difficulties in applying knowledge and skills to prospective nursing students due to a lack of competence, self-confidence, and feelings of inadequacy^(5-6)^.

The mental health of nursing students is also affected by increasing stress from the ever-increasing number of confirmed and suspected cases, media reporting in connection with the increase in COVID-19 cases, the feelings of not being sufficiently supported, inadequate application skills, uncertainty when, where, and how to conduct compensatory training, limited internet access and learning devices^(4,7-8)^. Additionally, anxiety among students during the pandemic has also been reported in several studies and is associated with fear of infection, concerns for the well-being of their families, unpreparedness for pandemic situations, and high morbidity rates due to COVID-19^(8-12)^.

Stress and anxiety in the learning environment require the utilization of coping mechanisms by nursing students^(13)^. A positive attitude correlated positively with emotional coping strategy is self-compassion, which involves being open and moved by one’s suffering, caring and being kind to oneself, understanding, not judging one’s shortcomings and failures, and recognizing that self-experience is part of the general human experience^(14-15)^. Additionally, self-compassion is important for students not only to cope with academic tasks and the pressures they experience during their education but also to develop their emotional abilities and skills in demonstrating compassion and caring for others^(16-17)^. Self-compassion was found to be able to alleviate perceived stress among nursing students and thereby reduce anxiety and depression^(18)^. This finding was further supported by another study conducted among nursing and medical students which found that self-compassion significantly affected the reduction of depressive symptoms by increasing resilience and optimism as well as reducing perceived stress^(19)^.

In light of this stressful time during the COVID-19 pandemic, self-compassion can serve as an adaptive emotional regulation strategy that involves treating oneself with a non-judgmental and caring approach oneself in the face of the challenges and stresses experienced by students. It can also be the best way to support nursing students in developing compassionate practices. An increased focus on self-compassion in nursing education offers possibilities for growth in developing compassionate practices.

## OBJECTIVE

To determine the level of self-compassion and investigate the relationships between sociodemographic variables and self-compassion in undergraduate nursing students at a state University in Indonesia during the COVID-19 Pandemic.

## METHODS

### Ethical considerations

Ethical approval was granted by the University Research Ethics Committee. Institution permission was obtained from the faculty in which the research was carried out. For the Indonesian version of the Self-Compassion Scale (SCS) used in this study, permission was obtained by e-mail correspondence with the author who also carried out the validity-reliability study of the Indonesian SCS^(20)^. Anonymity was maintained throughout. This study was voluntary and without any element of coercion, respondents can choose to agree or refuse to participate in this study.

### Design, period, and place of study

STROBE Statement was used for reporting this observational study^(21)^. This is a cross-sectional internet-based survey research. The data was collected through an online-based form from March 17 to April 10, 2022.

### Population and sample

The population selected were nursing students enrolled in the semester 2021/2022 academic year at a state University in West Java, Indonesia with a total of 799 students. Undergraduate nursing students from classes of 2018, 2019, 2020, and 2021 who were enrolled in the semester 2021/2022 academic year and any campus areas were eligible to participate. Additionally, 260 nursing students were recruited by using a proportionate stratified random sampling technique. The strata referred to classes of 2018, 2019, 2020, and 2021 and the campus areas.

### Procedure

#### Data collection tools

Data was collected using an online questionnaire consisting of two parts: a demographic questionnaire and a Self-Compassion Scale (SCS). Demographic questions such as age, gender, and ethnics were included. To measure self-compassion, this study used an Indonesian version of the SCS which consists of 26 items representing the 6 components of self-compassion: self-kindness, self-judgment, common humanity, isolation, mindfulness, and over-identification. The Indonesian version of SCS consists of five-point Likert items, ranging from 1 (‘Rarely’) to 5 (Almost always). Mean scores of each positive components (self-kindness, common humanity, and mindfulness) were calculated normally while mean scores of each negative components (self-judgment, isolation, and over-identification) were done after reverse coding. Total scores obtained by calculating the mean score of all the 6 components’ mean scores. Total scores ranging from 1.0-2.49 indicates low self-compassion, between 2.5-3.5 indicates moderate self-compassion, and 3.51-5.0 indicates high self-compassion.

#### Respondents Recruitment

The recruitment process of respondents began immediately after obtaining a Research Ethics Exemption and Institution Permission. Respondents were selected by their student ID number and divided by their classes and campus areas. The randomization was done using Microsoft Excel and list of student ID numbers who were selected as respondents was made into a new Microsoft Word document. Dissemination was carried out through broadcast to each class WhatsApp group with the help of each class president. To invite the selected students, the Microsoft Word document and link of the online form equipped with inform consent and research information was sent to each class WhatsApp group with the help of each class president. In this study, none of the respondents refused to participate. However, there were 79 substitute respondents who replaced respondents who did not respond to the personal follow-up that had been carried out. This can be explained further in data collection part.

#### Data collection

Data was collected through the Google Form^TM^ platform and opened for filling for three weeks. Before answering the questionnaire, respondents read and accepted the informed consent. ‘Required’ feature was activated in each of items of the Google Form^TM^ to anticipate missing answers. Respondents who had filled out the online form were recorded. A total of 167 respondents who were selected as respondents but had not filled out the online form for more than two weeks were followed up. Most respondents had not filled because they were in the exam week. 79 respondents who did not respond to the follow up were replaced by re-drawing the student ID number using Microsoft Excel. Personal follow-up was carried out until the number of samples is met. Then, the collected data was created in a separate Microsoft Excel document before processing.

### Analysis of results and statistics

This study used univariate and bivariate data analysis to reveal the frequency distribution of self-compassion among respondents and relationships between sociodemographic variables and self-compassion. Subsequently, categorical data for self-compassion were presented as frequency and percentage. Each of the SCS item average scores were presented by mean, standard deviation (SD), maximal and minimal values. Each component of self-compassion was analyzed to identify students’ tendency to respond to the Indonesian version of SCS items. Moreover, bivariate analysis was done using Spearman’s correlation.

## RESULTS

### Respondents’ characteristics

Based on Table 1, most of the respondents were female, between 19 - 23 years, and belonged to the group of young adults. More than half of the respondents’ ethnicity was Sundanese.

**Table 1:** Characteristics of Respondents (N=260)

Characteristic	Frequency (*f)*	Percent (%)
Gender		
Man	18	6.9
Woman	242	93.1
Age		
17 - 18 Years Old	40	15.4
19 - 23 Years Old	220	84.6
Ethnicity		
Sundanese	157	60.4
Javanese	73	28.1
Bali	3	1.2
Batak	7	2.7
Betawi	4	1.5
Minang	7	2.7
Others	9	3,5

### Self-compassion among Undergraduate Nursing Students at a State University in West Java, Indonesia

Based on Table 2, it was found that more than half of the students had a moderate level of self-compassion. There were a small number of students with low self-compassion and about a third of them had a high self-compassion.

**Table 2:** Self-compassion among Undergraduate Nursing Students during the COVID-19 Pandemic (N=260)

Variable	Category	Frequency (*f*)	Percent (%)
Self-compassion (Mean= 3.25; SD= 0.539)	Low	16	6.2
	Moderate	156	60
	High	88	33.8

Table 3 indicates the collective responses to each statement from the Indonesian version of the SCS. The components of self-compassion are divided into two types: positive and negative components. Additionally, the positive components of self-compassion consist of self-kindness, common humanity, and mindfulness, while the negative components consist of self-judgment, isolation, and overidentification. Within the positive component of self-compassion, an item with the highest mean value and often felt by respondents was “I try to be loving towards myself when I’m feeling emotional pain” which is one of the statements in the self-kindness component. While in the category of negative components, the item with the highest mean value and often felt by respondents was “When something upsets me I get carried away with my feelings” which is one of the statements on the over-identification component.

**Table 3:** Components of Self-compassion of Undergraduate Nursing Students during the COVID-19 Pandemic

Components	Items	Mean	SD	Min	Max
Positive Components					
Self-kindness	1. I try to be loving towards myself when I’m feeling emotional pain	^a)^3.93	1.016	1	5
	2. When I’m going through a very hard time, I give myself the caring and tenderness I need.	3.58	0.903	1	5
	3. I am kind to myself when experiencing suffering	^b)^3.31	0.981	1	5
	4. I’m tolerant of my own flaws and inadequacies.	3.54	0.902	1	5
	5. I try to be understanding and patient towards those aspects of my personality I don’t like	3.67	0.847	1	5
Common humanity	1. When things are going badly for me, I see the difficulties as part of life that everyone goes through.	^a)^3.86	0.887	1	5
	2. When I’m down, I remind myself that there are lots of other people in the world feeling like I am.	3.80	0.990	1	5
	3. When I feel inadequate in some way, I try to remind myself that feelings of inadequacy are shared by most people.	^b)^3.66	0.943	1	5
	4. I try to see my failings as part of the human condition	3.74	0.842	1	5
Mindfulness	1. When something upsets me I try to keep my emotions in balance.	^a)^3.82	0.936	1	5
	2. When something painful happens I try to take a balanced view of the situation.	3.62	0.859	1	5
	3. When I fail at something important to me I try to keep things in perspective.	^b)^3.34	1.055	1	5
	4. When I’m feeling down I try to approach my feelings with curiosity and openness.	3.56	0.914	1	5
Negative Components					
Self-judgment	1. I’m disapproving and judgmental about my own flaws and inadequacies	2.83	1.068	1	5
	2. When times are really difficult, I tend to be tough on myself.	3.38	1.138	1	5
	3. I’m intolerant and impatient towards those aspects of my personality I don’t like	2.91	1.125	1	5
	4. When I see aspects of myself that I don’t like, I get down on myself	^a)^3.45	1.037	1	5
	5. I can be a bit cold-hearted towards myself when I’m experiencing suffering	^b)^2.79	1.165	1	5
Isolation	1. When I think about my inadequacies, it tends to make me feel more separate and cut off from the rest of the world.	^b)^3.02	1.151	1	5
	2. When I’m feeling down, I tend to feel like most other people are probably happier than I am	^a)^3.46	1.092	1	5
	3. When I’m really struggling, I tend to feel like other people must be having an easier time of it	3.32	1.143	1	5
	4. When I fail at something that’s important to me, I tend to feel alone in my failure.	3.45	1.080	1	5
Overidentification	1. When I’m feeling down I tend to obsess and fixate on everything that’s wrong.	2.82	1.178	1	5
	2. When I fail at something important to me I become consumed by feelings of inadequacy.	3.13	1.109	1	5
	3. When something upsets me I get carried away with my feelings	^a)^3.58	0.941	1	5
	4. When something painful happens I tend to blow the incident out of proportion	^b)^2.66	1.088	1	5

a) Item with the highest mean value;

b) Item with the lowest mean value.

Based on gender, the proportion of female students with moderate self-compassion was higher than male students. On the other hand, the percentage of male students with high self-compassion was higher than that of females. It also shows that more than half of respondents within the adolescence and young adulthood groups had moderate self-compassion. Based on ethnicity, students with moderate self-compassion appear to be common across most ethnicities, except for Bataknese. Correlations between gender and self-compassion and ethnicity and self-compassion were all positive but not significant (p > 0.05). Meanwhile, there was a significant correlation between age and self-compassion among the undergraduate nursing students (p < 0.05) (Table 4).

**Table 4:** Undergraduate Nursing Students’ Self-Compassion based on characteristics

Characteristics		*Self-compassion*			*p*
		Low	Moderate	High	
Gender					0.845
Male	Frequency	3	8	7	
	Percentage	16.7%	44.4%	38.9%	
Female	Frequency	13	148	81	
	Percentage	5.4%	61.2%	33.5%	
Age					0.016^*^
17 - 18 Years Old	Frequency	8	22	10	
	Percentage	20%	55%	25%	
19 - 23 Years Old	Frequency	8	134	78	
	Percentage	3.6%	60.9%	35.5%	
Ethnicity					0.728
Sundanese	Frequency	7	97	53	
	Percentage	4.5%	61.8%	33.8%	
Javanese	Frequency	8	40	25	
	Percentage	11.0%	54.8%	34.2%	
Bali	Frequency	0	3	0	
	Percentage	0.0%	100.0%	0.0%	
Batak	Frequency	0	3	4	
	Percentage	0.0%	42.9%	57.1%	
Betawi	Frequency	0	2	2	
	Percentage	0%	50%	50%	
Minang	Frequency	1	3	3	
	Percentage	14.3%	42.9%	42.9%	
Others	Frequency	0	8	1	
	Percentage	0%	88.9%	11.1%	

* p < 0.05.

## DISCUSSION

### Self-compassion of Undergraduate Nursing Students at an Indonesian State University during the COVID-19 Pandemic

This study showed that there was a significant correlation between self-compassion and age but no significant correlation when it comes to gender and ethnicity. Furthermore, more than half of students had a moderate level of self-compassion. Nursing students’ self-compassion level was also found to be moderate in the previous studies^(22-23)^. The moderate level of self-compassion in nursing students may imply their ambiguous perceptions and feelings about their decision to act compassionately or uncompassionately. Individuals with moderate self-compassion are high in self-kindness, common humanity, and mindfulness but also high in self-judgment, isolation, and over-identification^(15)^. Students with moderate self-compassion are more likely to be self-compassionate on occasion rather than all of the time^(22)^. Nonetheless, people with moderate self-compassion had higher psychological well-being than individuals with low self-compassion on all psychological well-being measures (satisfaction, meaning of life, as well as resilience) except for the indicator of anxiety^(24)^. Furthermore, both individuals with moderate and high self-compassion used adaptive coping strategies (reassessment and positive reframing) and use fewer maladaptive coping strategies (rumination, self-blame, and repressing feelings).

Some students’ demographic variables were associated with self-compassion in this study. Particularly, there is a positive correlation between age and self-compassion. A possible explanation of this results is that self-compassion increases with age^(25)^. Baccalaureate nursing students were found to have adequate levels of self-compassion and self-care, but low levels of compassion^(26)^. Other finding suggested that older nursing students had more experience in a variety of work-related situations that can foster better relationships and promote compassion with patients and their families^(27)^. The results of the two studies indicated that self-compassion may increase as nursing students face various challenge in their life and self-compassion may also influence compassion they have for others, as stated in the recent study that self-compassion is associated with more compassion for others and self-compassionate people are more likely to provide social support and promote interpersonal trust^(28)^.

### Self-compassion of Undergraduate Nursing Students at an Indonesian State University during the COVID-19 Pandemic Based on Components

Students’ responses to self-compassion positive component items (self-kindness, common humanity, mindfulness) showed that they were quite able to be kind, warm, and understanding and able to empathize adequately with themselves in the face of suffering during the COVID-19 pandemic, quite aware that problems, failures, and difficulties are also experienced by other humans, and quite able to view the shortcomings, negative emotions, as well as bad experiences they encountered during the pandemic in a balanced and non-judgmental way. Previous evidence reported positive effect of self-kindness and common humanity on nursing students’ emotional intelligence and mental health^(18,29)^. This is because individuals with self-kindness can be gentle, supportive, and understanding towards oneself, accept oneself unconditionally and can comfort oneself actively in difficult times of COVID-19 pandemic, such as giving oneself space and time to rest emotionally or performing actions that support mental health such as engaging in positive self-talk, encouraging, and self-forgiving^(30-31)^. Furthermore, the COVID-19 situation may encourage students to comprehend that the pandemic is universal for all people and that all individuals are suffering changes in their respective lives as a result. By reminding oneself that others are experiencing the same, individuals will feel connected to others, and, the individual strengthens their sense of mutual belonging and social identity^(32)^. In addition, the uncertainty of the pandemic situation may allow students to understand the pandemic situation without allowing themselves to dissolve into emotions, and therefore they can quite clearly see what can be controlled and what cannot through mindfulness. This can be due to individual understanding of their suffering in mindful view and observing what happening (right here, right now)^(32-33)^.

Students’ responses to items that represented negative components of self-compassion (self-judgment, isolation, and over-identification) showed that students still tended to judge and criticize themselves when confronted with suffering during the COVID-19 pandemic, feel isolated and disconnected from other human beings when faced with suffering, deprivation, or failure during the pandemic, and obsess and continue to fixate on everything wrong when faced with problems. A negative and recurrent self-evaluation related to high self-criticism can lead to low self-esteem^(34)^. Self-esteem is important for nursing students as it helps them to have a better collaboration with colleagues and patients, and consequently, better performance later in the clinical/work setting^(35)^. Thus, low self-esteem can cause nursing students to have significant difficulties in communication with colleagues and patients. Moreover, when individuals feel detached from others, they believe that failure, feelings, and self-shaming are things that make them withdraw, hide their personality and emotions^(36)^. Showing emotions that do not match those observed is defined as a surface acting and is a destructive emotional strategy that causes cognitive and emotional fatigue^(37-38)^. Emotional fatigue was statistically and significantly correlated with compassion fatigue in nursing students^(39)^. Nursing students are at risk for compassion-related fatigue due to the consistent use of empathy in the clinical setting. The consequences of compassion fatigue can lead to ineffective patient care, increased medical errors, and increased stress^(40)^. Subsequently, students that are exposed to various negative emotions, for example, anxiety, systematically distort the meaning of events and therefore they interpret their experiences about an event in a negative and self-defeating way^(41)^. The overidentified individual understands that he or she feels down or suffering but dramatizes the situation at hand to the point where nothing else matters but their suffering^(32)^.

The result the current study suggests that self-compassion in undergraduate nursing students still need improvement because they still tend to criticize themselves, feel isolated, and ruminate about their problems. Three main strategies can support the development of self-compassion in undergraduate nursing students: compassion literacy, mindfulness training, and experiential exercises^(42)^. Compassion literacy provides insight into the skills that support compassionate delivery of care and self-care. It arises from the ability to know compassion and to be aware of the factors that hinder and support compassionate treatment^(43)^. Furthermore, mindfulness-based programs such as Compassionate mind training (CMT), Loving-kindness meditation (LKM), Mindful self-compassion (MSC), Mindfulness and acceptance-based (MAB), and Mindfulness-based compassionate living (MBCL) can support the development of mindfulness and self-compassion^(44-46)^. The integration of meditation-based strategies into the nursing curriculum is worth considering supporting the development of self-compassion and increasing the student’s self-confidence in providing compassionate care^(46)^. Developing self-compassion and compassion for others can also be encouraged by incorporating experiential compassion training, which can influence compassion^(47)^. Activities such as letter writing, reflection, meditation practice, and self-care activities to support meditation and self-compassion has shown an amplifying effect on self-compassion and compassion for others by impacting education awareness and mindfulness^(45)^. The integration of reflective activities and mindfulness-based experiences into the nursing curriculum aims to support the development of self-compassion and compassion for others in nursing students^(42)^.

Moreover, current study also highlighted the tendency of students to get carried away with feelings, which indicates that they still have difficulty managing their feelings or emotions. Difficulties in managing emotions in students are associated with procrastination that prevents them from achieving long-term goals, a decrease in academic performance, low achievement, and poor grades in college^(48-50)^. Emotional regulation workbooks can be developed to help students to recognize and regulate the emotions they are feeling. An adequate counselling services and regulation should also be accessible for students with various involvement and awareness of stakeholders in the campus environment, which includes leaders as policymakers, lecturers, academic supervisors, and students.

### Limitations

The first limitation of this study relates to the age range of respondents who belonged to the same group, which may limit the generalizability of this study. In addition, the mid-semester exam took place within a week during the data collection, which might affect the students’ responses. Moreover, the relationship between cultural aspects and self-compassion should be further explored when students come from different ethnic backgrounds, although they are dominated by Javanese and Sundanese. Another limitation of the study is the reduced frequency of the Bali and Betawi ethnic groups (with absolute frequencies lower than 5). In addition, the study was conducted after the critical period of the pandemic (March to April 2022) whereas the questions in the SCS were meant to be referred to how the students felt in the critical period of the pandemic.

### Contributions to the Field

The results are useful as information for the Faculty and University to develop self-compassion-based interventions or programs (intra-curricular, co-curricular, and extra-curricular) to improve emotional well-being in nursing students.

## CONCLUSION

During the COVID-19 pandemic, nursing students at an Indonesian State University were quite able to care for and understand themselves, comprehend that flawed experiences are shared by all humans, and offer clear and balanced attention to negative experiences. A positive and significant relationship was found between age and self-compassion, indicating that self-compassion may increase with age. It is suggested that compassion-related literacy, mindfulness training, and compassion-based experiential exercises be embedded into the current curriculum to support the development of self-compassion and compassion for others in nursing students, as well as increase the confidence of students in providing compassionate care. In addition, to reduce the level of overidentification of students, the faculty is expected to be able to provide student counseling services adequately, organizing emotional regulation programs such as workshops and developing workbooks or modules for the students.

Further study can be conducted with a wider scope of the population or different students’ characteristics. A prospective study could also identify predictors of the self-compassion of undergraduate nursing students.
